# Galacto-Oligosaccharides and the Elderly Gut: Implications for Immune Restoration and Health

**DOI:** 10.1016/j.advnut.2024.100263

**Published:** 2024-06-17

**Authors:** Yunan Hu, Mashael R Aljumaah, Maria Andrea Azcarate-Peril

**Affiliations:** 1 Department of Nutrition, Gillings School of Global Public Health, University of North Carolina, Chapel Hill, NC, United States; 2 UNC Microbiome Core, Center for Gastrointestinal Biology and Disease (CGIBD), School of Medicine, University of North Carolina, Chapel Hill, NC, United States; 3 Department of Plant and Microbial Biology, North Carolina State University, Raleigh, NC, United States; 4 Department of Botany and Microbiology, College of Science, King Saud University, Riyadh, Saudi Arabia

**Keywords:** galacto-oligosaccharides, immunosenescence, short-chain fatty acid

## Abstract

The increasing prevalence of noncommunicable diseases in the aging population has been correlated with a decline in innate and adaptive immune responses; hence, it is imperative to identify approaches to improve immune function, prevent related disorders, and reduce or treat age-associated health complications. Prebiotic supplementation is a promising approach to modulate the gut microbiome and immune system, offering a potential strategy to maintain the integrity of immune function in older individuals. This review summarizes the current research on prebiotic galacto-oligosaccharide (GOS) immunomodulatory mechanisms mediated by bacterial-derived metabolites, including short-chain fatty acids and secondary bile acids, to maintain immune homeostasis. The potential applications of GOS as immunotherapy for age-related disease prevention in older individuals are also highlighted. This aligns with the global shift toward proactive healthcare and emphasizes the significance of early intervention in directing an individual’s health trajectory.

## Highlights

1. Life expectancy is increasing, and changes in immunity and gut microbiota occur with aging, leading to age-related diseases associated with immunosenescence.
2. GOS are one of the most common and widely known types of nondigestible prebiotics that significantly impact host health, demonstrating promising application efficacy in older individuals.
3. GOS immunomodulates immune homeostasis by modulating the fragile microbiota in older individuals to prevent or alleviate immune-related diseases.
4. SCFAs metabolism and secondary bile acid metabolism activated by GOS-derived bacteria plays a vital role in the immune system.
5. The clinical application of GOS in older individuals requires further study; however, source-specific and structure-specific differences have been noted.

**Statement of Significance**

This review provides compelling evidence that galacto-oligosaccharides (GOSs), as a dietary intervention, can significantly enhance gut health and immunomodulation in older adults. Based on these findings, the review urges further research to advance our comprehension of GOSs and their potential to optimize the health of older individuals.

## Introduction

As life expectancy increases, the global aging population is expanding at an unprecedented rate. According to the United Nations, in 2020, the global population aged ≥65 y was 800 million. By 2050, this number is projected to double to 1.55 billion, and the aging population will be twice the number of children aged <5 y and almost equivalent to the number of children <12 y. Moreover, the number of people aged ≥80 y are expected to triple between 2020 and 2050, reaching 426 million [1]. With advancing age, multifaceted changes in the immune system emerge, a process termed immunosenescence, which is characterized by a reduction in immune responsiveness, a decrease in mucosal resistance and barrier function, and an increase in susceptibility to infections [2]. Older adults are at risk of a myriad of age-associated diseases, such as chronic respiratory and kidney diseases, diabetes mellitus, and other noncommunicable diseases, which are primarily associated with chronic systemic inflammation (“inflammaging”) [3]. A clear event that highlighted the deterioration of the immune system in older adults was the COVID-19 pandemic, which presented an extraordinary challenge to public health and emphasized the vulnerability of the aging population to emerging diseases [4]. According to the United States Centers for Disease Control and Prevention [5], the hospitalization rate for COVID-19 of individuals aged ≥85 y was 10 times higher than that of 18- to 29-y-olds, while the rate of COVID-19-related deaths for those aged 65 to 74 y was 65 times higher than that for the 18- to 29-y-old group.

A key player in the decline in innate and adaptive immune responses is the gut microbiome, which significantly influences the development and progression of systemic inflammation, exacerbating or mitigating age-related effects on immune function. Therefore, a thorough understanding of the gut–microbiome–immune axis is vital as it represents a critical component of effective strategies to enhance immune function, prevent related disorders, and manage or treat health complications associated with aging.

Prebiotics and probiotics are important dietary supplements that could be effective in promoting health among the elderly by modulating the gut microbiota community and regulating the gut environment, including the immune system [6,7]. A randomized clinical trial showed that prebiotics (soluble corn fiber) combined with probiotics enhanced innate immunity by increasing natural killer (NK) cell activity in the elderly population [8]. Similarly, the prebiotics inulin plus fructo-oligosaccharides (FOSs) have been shown to mitigate frailty syndrome in the elderly [9], and nondigestible polysaccharides have been shown to enhance the immune response and increase the seroprotection rate for virus infection in the elderly [10]. Compared to infants, the study of prebiotics’ effects in adults and older adults is relatively underexplored. As research on prebiotics’ effects on the gut microbiome and the immune system advances toward understanding the effects of pure compounds, the existing literature must be compiled and revised to assist in the experimental design of future clinical trials. GOS has been studied for decades, with emphasis on its beneficial effects as a dietary intervention for infants [11]. However, it is only in the last decade that pure GOS with <10% free sugars (lactose, glucose, galactose) has been evaluated in clinical trials [[12], [13], [14], [15]]. Additionally, research on the effects of GOS on the elderly population is limited, with only 2 clinical trials to date solely focused on the elderly [16,17]. This review aims to bridge this gap by inferring potential effects from existing research on younger populations. We propose that the insights gained from studies on infants and adults can help explore GOS as a promising approach for the elderly. We aim to emphasize the potential benefits of GOS and encourage further research in this direction, adding a new perspective to the existing knowledge on GOS and its applications. In this review, we will discuss the rationale and potential advantages of using GOS as a prebiotic supplement to improve gut health in older adults.

## Immune System Alterations Associated with Aging

**FIGURE 1:**
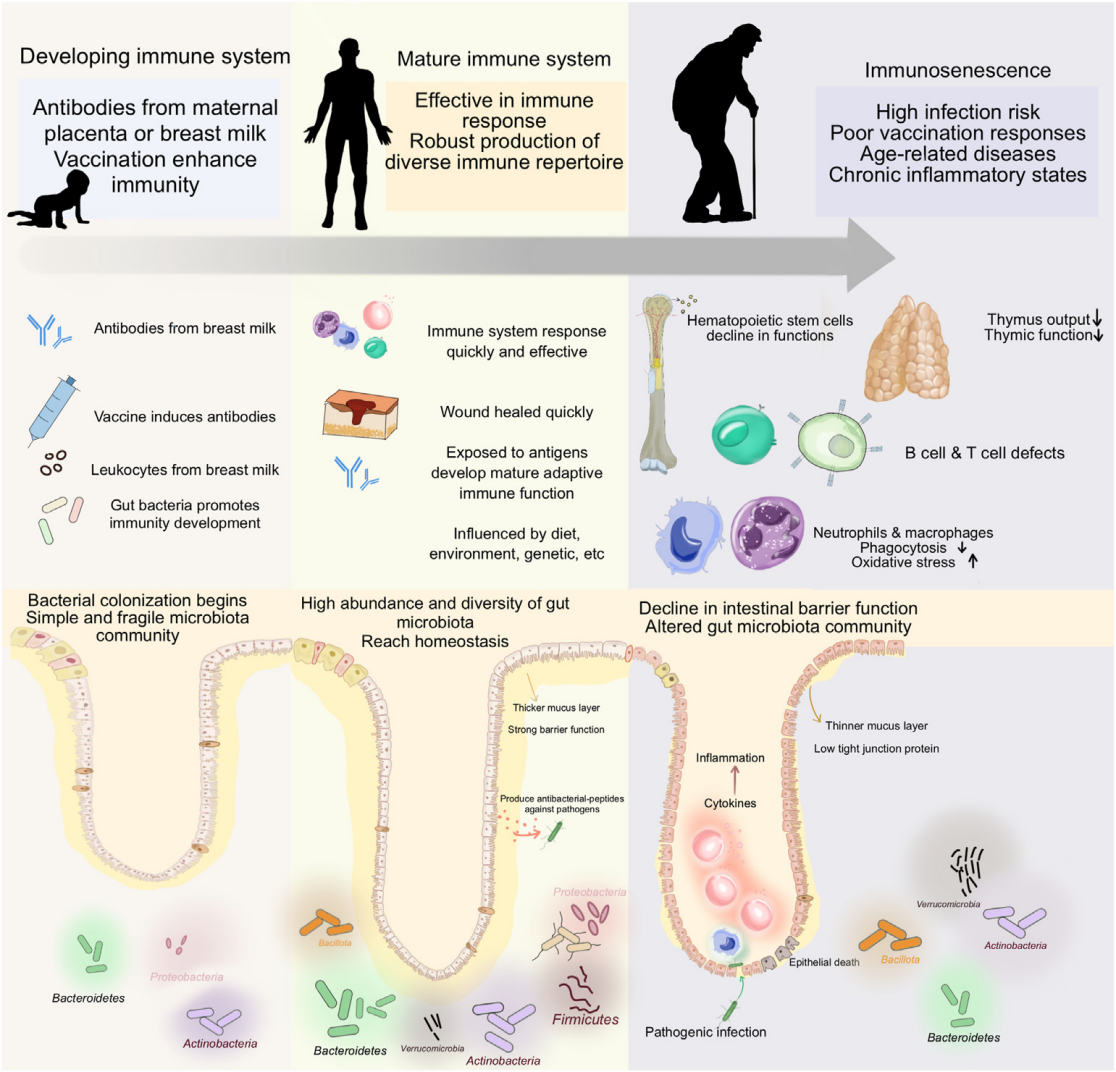
Comparison of the gut microbiota profile and immune function between children, young adults, and older individuals.

Innate and adaptive immunities become impaired with age (Figure 1). It is well established that, in older adults, there is a decline in the number and function of innate immune cells, leading to impaired pathogen elimination and chronic low-grade inflammation, characterized by increased proinflammatory cytokines [18,19]. The gastrointestinal tract, the largest immune organ, plays a crucial role in immune homeostasis, mediated by intestinal stem cells (ISCs) and intestinal epithelial cells (IECs), which maintain intestinal integrity and facilitate immune responses [20]. Aging adversely affects ISCs, IECs, and the structure of the intestinal crypts and villi, impairing regeneration and immune signaling. Metabolites such as MUFAs, PUFAs, N-acyl ethanolamines, and branched-chain amino acids are linked to longevity and improved immune homeostasis in the elderly [21]. This complex interplay of cells and metabolites underscores the multifaceted nature of immune regulation during aging.

## Microbial Shifts and Maturation of Immune Function

The evolution of the composition and functionality of the gut microbiota are significantly associated with immune function [[22], [23], [24]]. Establishment of the gut microbiome begins at birth. Assembly of the infant’s gut microbiome is influenced by delivery mode, the environment, and feeding type, as recently reviewed by Korpela et al. [25]. It is widely recognized that the early gut microbiota of vaginal-born, breastfed infants is dominated by *Bifidobacterium* and *Bacteroides* species. In contrast, babies born by cesarean section are dominated by *Enterobacter* with a high abundance of *Clostridium* in the first week of life [26,27]. Breastmilk delivers maternal soluble factors, such as macromolecules, immunoglobulins, cytokines, and immunologically active milk cells to the infant, which promote the maturation of the immune system [28]. Breastfeeding also introduces the microbes that colonize the infant’s gut and contribute to the development of the microbiota community during the first year, increasing the abundance of *Bifidobacterium*, *Akkermansia*, and *Bacteroides* [29,30]. Prolonged breastfeeding, beyond 6 mo, has been linked to the optimal maturation of the immune system [31] and a reduction of the intestinal dysbiosis associated with diarrhea [32,33]. Just as the infant gut microbiome, which exhibits high variability and low diversity until ∼3 y, the infant’s immune system is also immature and constantly dynamic, undergoing significant changes in the first year of life [25]. In infants, the balance between proinflammatory and anti-inflammatory cytokines is crucial to the appropriate response to pathogens and the development of early immune tolerance [31].

For the adult gut microbiome profile, there is yet to be a consensus on the definition of a healthy gut microbiome, given its susceptibility to be influenced by factors such as geographic location, living conditions, and socioeconomic status. For example, *Bacteroides* are dominant in people from China (39%), United States (38%), and Spain (23%) compared to those from Peru (1%), Malawi (3%), and Japan (5%) [[35], [36], [37]]. The considerable ecological diversity present in the gut microbiomes of healthy adults makes it exceptionally challenging to define a universal “healthy microbiome” at the population level. However, at the individual level, the characteristics and structure of the gut microbiota tend to remain relatively stable throughout adulthood. Dietary changes or medications can lead to short-term alterations, followed by a rapid restoration to a baseline gut microbiota [[38], [39], [40]]. This resilience of the gut microbiota complements the mature and robust immune system of healthy adults, which is more developed and better equipped to mount an effective immune response compared to that of infants and the elderly [41,42].

With advancing age, it is common to observe shifts in the composition of the gut microbiota and alterations in the immune system [21,43,44]. A detailed discussion of the interactions between specific taxa and the immune system is presented elsewhere; however, a common trend observed in aging is a reduction in the diversity and abundance of beneficial microorganisms, which can lead to the dysregulation of gut homeostasis and the immune system. The older adult gut microbiome is generally dominated by *Bacteroidota* and *Bacillota* [26]. *Actinomycetota*, *Pseudomonadota*, and *Verrucomicrobiota* have also been reported in high abundance [26,45,46]. An increase in the relative abundance of *Bacteroidota* and *Pseudomonadota* has been observed in subjects aged >70 y compared to the adult population (<60 y) [35], while the relative abundance of *Akkermansia* and *Bifidobacterium* has been reported to increase among Super Agers (ages 105–109 y) [47]. However, studies have shown that the diversity within taxa of *Bacteroides*, *Clostridium* cluster XIVa, *Bifidobacterium,* and *Faecalibacterium* species is lower in older adults (ages 60–80 y) [35,48]. Additionally, *Bacteroidaceae*, *Lachnospiraceae,* and *Ruminococcaceae* showed negative associations with aging, while *Eggerthella*, *Akkermansia*, *Anaerotruncus*, and *Bilophila* have positive associations with aging [26,35,49]. Over time, compositional changes in the microbiota are reflected in its metabolic function. For example, *Clostridium* cluster XIVa is associated with short-chain fatty acid (SCFA) production and maintenance of immune homeostasis [50,51]. The abundance of *Clostridium* cluster XIVa decreases significantly with aging, leading to a decrease in SCFA production [48,52].

A new and exciting possibility for maintaining a youthful and healthy immune system is to modulate the gut microbiome in a controlled, systematic, and personalized manner. This is made possible by the wealth of information provided by high-throughput “omics” technologies. It is well established that antibiotics, diet, and lifestyle influence gut microbial communities [53,54]. The acknowledgment of probiotics as positive modulators of gut health, including the immune system, can be traced back to the early 1900s with the work of Elie Metchnikoff [55]. Similarly, prebiotics have demonstrated effective modulation of the gut microbiota and have shown promise in the elderly demographic.

## Galacto-oligosaccharides

Prebiotics including GOSs, inulin, and FOSs, as well as human milk oligosaccharides and starch-derived oligosaccharides such as resistant starch and polydextrose, have been shown to confer multiple health benefits [[56], [57], [58]]. These benefits include enhancing the growth of beneficial bacteria such as *Bifidobacterium* (bifidogenic effect), improving digestive health, managing blood sugars and lipids, modifying immune markers, and providing anti-inflammatory effects [58,59]. GOS, a well-known type of nondigestible oligosaccharide, particularly benefits gut health by increasing the abundance of *Bifidobacterium*, *Akkermansia*, and *Bacteroides* [60,61]. They have an extensive history of safety and have been used for decades to improve overall host health via modulation of the gut microbiota [62]. GOSs generally contain a mixture of oligosaccharides with different polymerization degrees and linkages and are synthesized using microbial or enzymatic methods [11], often involving β-galactosidases from sources such as *Kluyveromyces fragilis*, *Aspergillus niger*, and *Lactobacillus reuteri* [63,64]. During the conversion from lactose to GOS, a range of mono-, di-, and oligosaccharides is created [64]. Commercial GOSs, such as Vivinal GOS, Bimuno, and Oligomate, vary in composition, impacting study reproducibility and outcome predictability. To tackle this issue, our group has cloned and heterologously expressed the β-hexosyltransferase from *Hamamotoa singularis* to generate high-purity (>90%) GOS [[64], [65], [66]] and lactosamine-enriched GOS [67].

Selectivity is an essential property of GOS as a prebiotic. Within this context, selectivity pertains to the notion that exclusive beneficial bacterial strains, notably lactobacilli and bifidobacteria, can metabolize GOS as a primary carbon source [56]. Consequently, GOSs increase the abundance of specific primary and secondary degraders, expanding autochthonous beneficial members of the intestinal microbiota [68]. It is well known that GOSs increase the abundance of *Bifidobacterium* (the “bifidogenic effect”), which is fundamental to the beneficial effect of the prebiotic and can lead to increased concentrations of colonic SCFAs, particularly butyrate [69]. By increasing the abundance of beneficial bacteria, GOSs limit the growth of pathogens, such as inhibition of the growth of *Staphylococcus aureus* and *Pseudomonas aeruginosa* in vitro [70] and inhibition of *Escherichia coli* E2348/69 adherence ex vivo [71]. Another factor contributing to selectivity is the glycosidic linkages and molecular weight of GOS components. It has been proposed that oligosaccharides of higher molecular weight may enhance saccharolytic fermentation in the distal colon to prevent chronic conditions [62].

Although GOS has been shown to beneficially modulate the gut microbiota and immune system, not everyone responds to GOS similarly (responders and nonresponders). Individuals can respond differently to GOS based on their dietary habits, microbiome, and genetics [56,72]. A recent clinical trial using GOS, dextrin, and inulin to investigate the responder mechanism of prebiotics revealed that the baseline gut microbiota profiles and metabolite composition were correlated with the participant response rate [72]. When comparing the initial SCFA concentration in the participants’ fecal samples and their response to prebiotics after 6 wk, those with the highest initial proportional butyrate concentrations had the weakest butyrogenic prebiotic reactions. This finding suggests an inverse relationship between the prebiotic response and baseline fecal SCFA concentration. Older adults with lower butyrate concentrations may have higher GOS response rates than young adults.

Currently, GOSs are approved by the United States Food and Drug Administration for supplementation at a concentration of ≤7.8 g/L or 11 g/serving for use in term infant formula and selected foods and beverages. GOS preparations have 14 Generally Recognized As Safe notifications. However, more research is needed on the recommended supplementation dosage for older adults and the specific type of GOS, its purity level, and its structural characteristics that would impact older individuals’ health.

## Immune Modulation of GOS Mediated by Beneficial Bacteria

**FIGURE 2:**
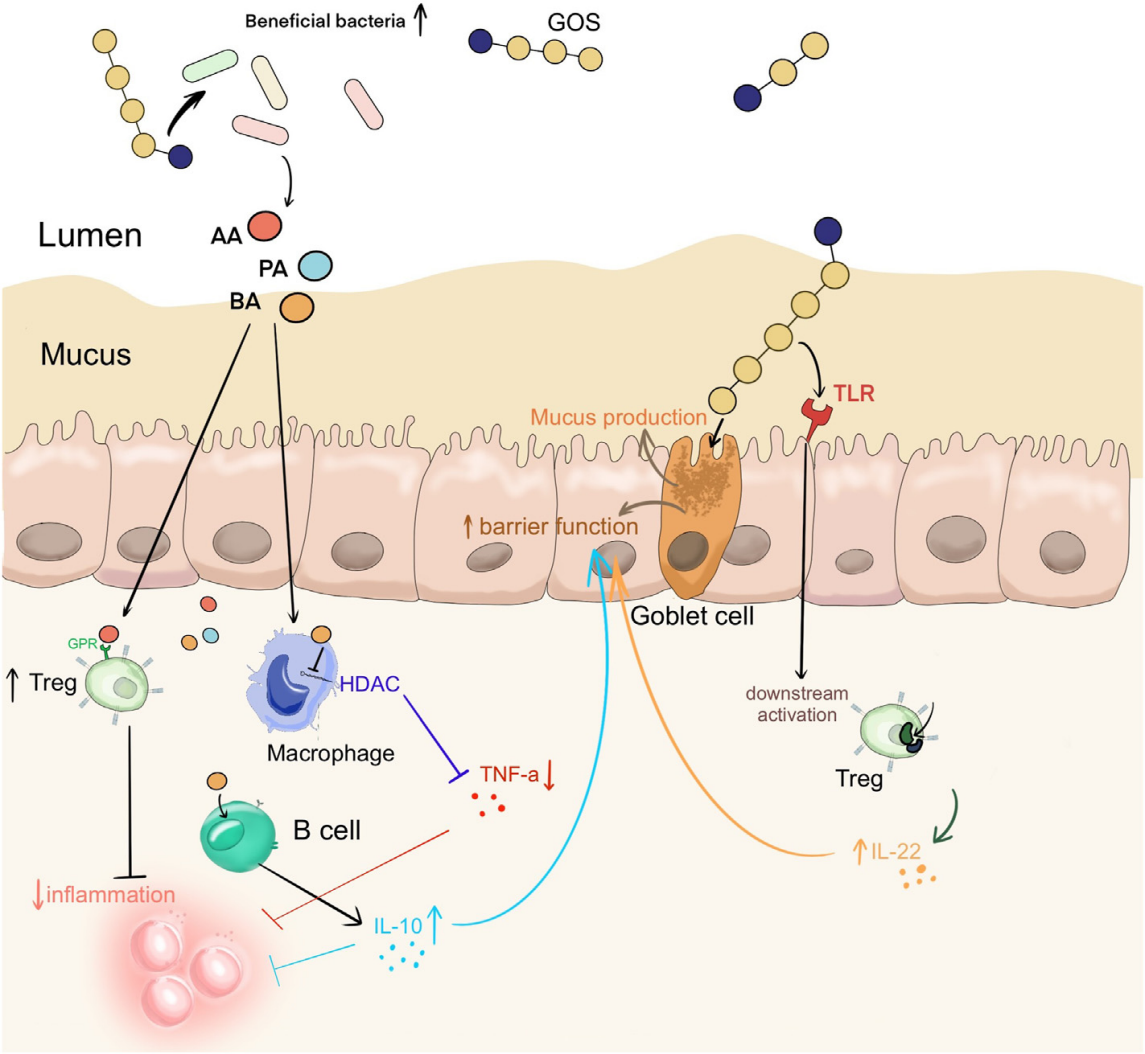
Immunomodulatory functions of GOS. AA, acetic acid; BA, butyric acid; GOS, galacto-oligosaccharide; GPR, G-protein-coupled receptor; HDAC, histone deacetylase; IL, interleukin; PA, propionic acid; TLR, Toll-like receptor; TNF, tumor necrosis factor; Treg, regulatory T cell.

The primary mechanism by which prebiotics modulate immunity is through the enhancement of beneficial bacteria (Figure 2). In adults, GOSs increase the abundance of gut-beneficial taxa, including *Bifidobacterium*, *Lactobacillus*, and *Akkermansia* [12,14]. Likewise, studies on older individuals showed that GOS increased the abundance of *Bifidobacterium*, *Lactobacillus-Enterococcus* spp., and *Clostridium coccoides–Eubacterium rectale* [16,[73], [74], [75]]. The increased abundance of *Bifidobacterium* has been associated with improved immunological function and protection against inflammatory and infectious diseases, such as irritable bowel syndrome (IBS), pouchitis, and *Clostridium difficile* infection [76]. Moreover, *Bifidobacterium* has been shown to enhance the biosynthesis of immunomodulatory molecules such as tryptophan and exopolysaccharides, which stimulate the activity of immune cells, promoting the generation of the cytokine IL-10 [77,78]. Additionally, it has been reported that *Bifidobacterium* species induce dendritic cell maturation and promote T cell polarization response [79].

Although the bifidogenic effects of GOS are well established, research regarding other beneficial microbes enhanced by GOS, including *Akkermansia muciniphila*, is lacking. *A. muciniphila*, a highly abundant bacterium in the intestinal mucus layer, has attracted significant attention for its beneficial effects against chronic diseases, including inflammatory bowel disease (IBD), cancer, diabetes, and obesity [80]. The abundance of *A. muciniphila* and acetic acid levels decreases with aging [81]. In mouse models of aging, *A. muciniphila* induced the adaptive immune response by producing SCFAs, increased mucin production enhancing intestinal barrier integrity [82,83], and reduced levels of proinflammatory cytokines, alleviating lymphocyte infiltration in ileum tissue [84]. SCFAs are the most important and well-studied microbial metabolites, and their beneficial effects have been studied extensively [85,86]. SCFA concentrations show a consistent age-related decline, with the concentration in those aged >80 y being less than half that of younger adults (<50 y), which correlates with the evolution of the gut microbiome as we age [48,87].

Bile acids, also transformed by gut bacteria such as *Bacteroides* and *Lactobacillus*, play key roles in host metabolism, cancer progression, and immunity (reviewed in [[88], [89], [90]]). These transformations include deconjugation and dehydroxylation, impacting the signaling properties of bile acids. Evidence suggested a positive correlation between GOS and secondary bile acid metabolism. In a study on rats, administering GOS (800 mg/kg) once a day significantly increased bile salt hydrolase activity in the small intestine, as indicated by serum biochemical parameters [91]. A mouse study showed that GOS modified bile acid metabolism by regulating the intestinal microbiota, translating into a protective effect against Pb toxicity [92]. Notably, for the population aged >100 y, their gut microbiome profile is enriched in microorganisms that can generate unique secondary bile acids [93]. Therefore, the production of secondary bile acids may contribute to healthy aging.

## Enhancing the immune system through direct interaction with GOS

Toll-like receptors (TLRs) are essential receptors of the innate immune system that recognize pathogen-associated molecular patterns and initiate immune responses [94]. TLRs, expressed in immune cells, such as antigen-presenting and IECs, activate downstream signaling pathways [95]. TLR4 has been reported to bind to GOS and other oligosaccharides and trigger immune responses that promote the development of a healthy gut microbiome and enhance host immunity [96]. Additionally, Sun et al. [97] reported that GOS reduced LPS-induced inflammation in macrophages through TLR4, exerting anti-inflammatory activity. GOS has also been shown to reduce pathogen bacterial viability via the TLR4/NF-κB pathway and can relieve lung infections and inflammation [98]. TLRs are essential in promoting a network between the gut microbiota, IECs, and the immune system, underpinning the immunomodulatory ability of GOS in humans.

In addition to TLR, GOSs can directly modulate the immune system through additional pathways. In Caco-2 cells, GOS inhibited the NF-κB pathway induced by dextran sulfate sodium salt, thereby counteracting the inflammatory state of the host [99]. In LS174T cells, GOS modulated the function of intestinal goblet cells and improved mucus barrier function by acting directly on the barrier function-related genes *MUC2*, *TFF3*, and *RETNLB* [61,100].

## Strengthening the Aging Gut Barrier: the Impact of GOS on Intestinal Permeability

Intestinal barrier function is a significant indicator of gut homeostasis and a factor that drives immunosenescence [101]. With aging, the integrity of the mucus layer deteriorates, leading to a decline in its antimicrobial functionality. This decline can be attributed to various factors, such as changes in mucin production by a reduction in the number of goblet cells, alterations in the composition of the gut microbiota, and an overall diminished efficiency of the immune system due to aging [61,102,103]. These changes can lead to a phenomenon known as “intestinal barrier dysfunction,” where the protective barrier becomes more permeable. The mucus layer loses its antimicrobial function, thus allowing potentially pathogenic bacteria, viruses, and harmful metabolites to pass through the intestinal barrier and into the bloodstream. This increase in permeability is often referred to as “leaky gut” and occurs in older individuals with intestinal inflammation [104]. Under normal conditions, a well-regulated process maintains gut mucosal tolerance by controlling the trafficking of antigens in the body. However, gut dysbiosis disrupts this balance, weakening the gut barrier and allowing harmful substances from the gut to enter the bloodstream, triggering inflammation. This contributes to the development of chronic inflammatory diseases by triggering the release of proinflammatory cytokines, including TNF-α and interferon-γ, which further exacerbate the condition by increasing the permeability of the paracellular pathway for antigen passage, thereby creating a cycle of inflammation [101]. Numerous studies in animal models and humans have established a link between gut permeability, noninfective chronic inflammation, and metabolic changes commonly associated with aging [[105], [106], [107], [108]].

The notion of GOS as a modulator of intestinal barrier function is a recent development. Evidence suggests that GOS can reduce intestinal permeability of the inflamed gut. Studies of aged mice (>60 wk) examining the effects of GOS on the aging gut showed that GOS reduced the age-associated compromised intestinal permeability and increased *MUC2* expression and mucus thickness [61,100]. In a study in Caco-2 cells, GOS had a protective effect on the monolayer’s permeability when disrupted by mycotoxin deoxynivalenol [33]. This suggests that GOS could play a significant role in strengthening the aging gut barrier and maintaining gut health in the elderly.

## GOS-Induced Immunomodulation in the Elderly

The immunomodulatory effects of GOS are well established in infants and young populations; however, the mechanisms involved remain unclear [11,109,110]. Although less explored in older adults, one study showed that GOS increased phagocytosis and NK cell activity while decreasing the production of proinflammatory cytokines, including IL-6, IL-1β, and TNF-α, and increasing the production of the anti-inflammatory cytokine IL-10 in older individuals (aged 69.3 ± 4.0 y) [17]. In another study, GOS increased the abundance of *Bacteroides* and *Bifidobacterium* in elderly individuals (aged 65–80 y). This increase was associated with higher concentrations of lactic acid in fecal samples [16]. This study also revealed enhanced immunity, characterized by elevated IL-10, IL-8, NK cell activity, C-reactive protein levels, and reduced IL-1β levels.

Although most studies on prebiotics and probiotics in older adults have shown positive impacts (Table 1 [9,17,[111], [112], [113], [114], [115], [116], [117]]), certain subgroups, such as those suffering from IBS or IBD, those recovering from viral infections, and specific vaccine cohorts, are underrepresented in this research. It is worth noting that older adults respond differently to interventions. Although some experience positive results, there is a group of individuals who do not respond. This could be due to various factors including the dosage of prebiotics, their initial gut microbiome, lack of adherence to the intervention, and the duration of the intervention itself. All these elements can impact the effectiveness of the intervention. Reported interventions lasted from 3 d [117] to 28 wk [9], and the dosages ranged from 1.3 g/d [113] to 10 g/d [117]. The outcomes of those studies also vary; some showed positive effects on improving exhaustion, lowering potential mortality, reducing inflammation, enhancing SCFA production, and increasing the abundance of beneficial bacteria [17, 111,117, 114], while others showed either placebo effects or no significant changes [113,112–116. There was no obvious association between dosage, duration, or population age and the outcomes. This variability underscores the necessity for further research to understand the underlying mechanisms that influence these differential responses.

**TABLE 1:** Summary of health effects of prebiotics in the older population

Reference	Intervention	Population	Health condition	Study design	Main health outcome	Main findings	Potential implications
Walton et al. [111]	4 g GOS and placebo twice a day for 3 wk, preceded by 3-wk washout period	39 volunteers aged 50–81 y	Healthy	Randomized, double-blind, placebo-controlled crossover trial	1) Identify and quantify the bacteria in fecal sample 2) SCFA analysis 3) Multiple-stage continuous culture of fecal water	Significant bifidogenic effects. A significant increase in the amount of butyrate.	GOS can be utilized as a selective ingredient to positively influence the composition of beneficial gut bacteria and mitigate the risk factors related to changes in the colonic microbiota and fermentation that occur with aging.
Vulevic et al. [17]	5.5 g/d GOS for 10 wk, preceded by 4-wk washout period	44 volunteers aged 69.3 ± 4.0 y	Healthy	Randomized, double-blind, placebo-controlled, crossover study	1) Predominant bacterial groups were quantified 2) Phagocytosis, NK cell activity, cytokine production 3) Plasma cholesterol, and HDL cholesterol	Significantly increased the numbers of beneficial bacteria, especially bifidobacterial. Significant increases in phagocytosis, NK cell activity, and the production of anti-inflammatory cytokine IL-10. Significant reduction in the production of proinflammatory cytokines.	B-GOS administration to healthy elderly persons resulted in positive effects on both the microflora composition and the immune response.
Bunout et al. [112]	6 g/d mixture of 70% raftilose and 30% raftiline for 28 wk	66 volunteers aged ≥70 y	Healthy	RCT	1) The level of serum total proteins, albumin, immunoglobulins, saliva secretory IgA, and serum titers of influenza A and B and pneumococcal antibodies 2) At wk 8, cultured peripheral monocyte cells, secretion of IL-4, IFN-γ, and lymphocyte proliferation, stimulated with phytohemagglutinin and influenza antigen, were measured	No changes in serum proteins, albumin, Igs, and secretory IgA were observed. Antibodies against influenza B and pneumococcus increased significantly from wk 0 to 8, with no significant differences between groups. Antibodies against influenza A did not increase. No effects of prebiotics on IL-4 and IFN-γ secretion by cultured monocytes were observed.	No immunological effects of the mixture of raftilose and raftiline response to vaccination in the elderly were observed in this study.
Schiffrin et al. [113]	1.3 g/d FOS for 12 wk	74 volunteers aged 70–99 y	Malnourished or at risk of malnutrition	Prospective, randomized, double-blind, controlled study	1) Nutritional evaluation, serum immunoglobulins, lymphocyte subsets, various cytokines and the endotoxin sCD14 in serum 2) Cytokines specific mRNA in peripheral blood mononuclear cells at baseline and 12 wk 3) Fecal bacteriology	Specific mRNA extracted from blood leucocytes showed a different level of proinflammatory gene activation; Serum levels of sCD14, a product shed by activated macrophages, decreased only in the OS group without reaching statistical significance. No significant differences were detected in the fecal gut flora or in the nutritional parameters.	The administration of supplements in older persons at risk of malnutrition may benefit from the addition of prebiotics that can improve the low noise inflammatory process frequently observed in this population.
Buigues et al. [9]	Mixture of inulin (3375 mg) and FOS (3488 mg) once daily	60 volunteers aged 66–90 y	Mobile, non-demented nursing home residents	Randomized, double-blind, placebo-controlled trial	1) Frailty criteria 2) Hemogram and biochemical blood analysis	Significantly improved exhaustion and handgrip strength; no significant differences in the serum concentration of TNF-α.	The overall rate of frailty was not significantly modified by prebiotics.
Jain et al. [114]	Synbiotics of *Lactobacillus acidophilus* LA5, *Bifidobacterium lactis* Bb 12, *Streptococcus thermophilus*, and *Lactobacillus bulgaricus* with oligofructose, once daily until death or discharge from current hospital	90 volunteers aged 62–80 y	Patient admitted to an ICU	RCT	Gut permeability and barrier function	After 1 wk of therapy, patients in the synbiotic group had a significantly lower incidence of potentially pathogenic bacteria and multiple organisms in their nasogastric aspirates than controls. There were no significant differences between the groups in terms of intestinal permeability, septic complications, or mortality.	Synbiotics altered the microbial composition of the upper gastrointestinal tract but had no effect on intestinal permeability.
Louzada et al. [115]	Synbiotics of *Lactobacillus paracasei*, *Lactobacillus ramnosus*, *Lactobaciullus acidophilus*, *Bifidobacterium lactis* (108–109 CFU of each) with FOS (6 g), twice daily for 24 wk	49 volunteers aged 65–90 y	Prefrail individuals	RCT	GDS-15; MMSE; body fat percentage; serum IL-6, TNF-α, and IL-10; serum DAO, IFABP, and LPS	Both placebo and synbiotics groups had reduced body fat percentage, TNF-α, and DAO. IL-10 was significantly increased only in the synbiotics group.	Synbiotics had weak effect on depressive symptoms and more optimistic effects on cognition in apparently healthy elderly.
Neto et al. [116]	Synbiotics of 6 g FOS, 10^8^ to 10^9^ CFU *Lactobacillus paracasei*, *Lactobacillus rhamnosus*, *Lactobacillus acidophilus*, and *Bifidobacterium lactis*, once daily dose for 3 mo	17 volunteers aged 60–74 y	Community dwelling older adults fulfilling one of Fried’s frailty criteria	Double-blind, placebo-controlled study	Anthropometric measurements, BIVA, IL-6, and TNF-α	Majority of synbiotics individuals maintained or improved their tissue hydration.	3 mo of synbiotic supplementation did not promote any significant changes in inflammatory cytokines or body composition but demonstrated a trend toward a preservation of hydration status in apparently healthy elderly individuals.
Shimizu et al. [117]	Synbiotics of 3 × 10^8^*Bifidobacterium breve* strain Yakult, 3 × 10^8^*Lactobacillus casei* strain Shirota, and 10 g GOS, once daily dose for 3 d	72 volunteers aged 64–82 y	Septic patients placed on a ventilator within 3 days of ICU admission	Single-blind study	1) Infectious complications including enteritis, VAP, and bacteremia within 4 wk from admission 2) Mortality, fecal bacterial counts, and organic acid concentration within 4 wk	The incidence of enteritis and incidence of VAP was significantly lower in the Synbiotics than the No-Synbiotics group; total organic acid concentration, especially the amounts of acetate, were significantly greater in the Synbiotics group than in the No-Synbiotics group at the first week.	Prophylactic synbiotics could modulate the gut microbiota and environment and may have preventive effects on the incidence of enteritis and VAP in patients with sepsis.

Abbreviations: BIVA, bioelectric impedance with vectorial analysis; CFU, colony-forming units; DAO, diamine oxidase; FOS, fructo-oligosaccharide; GDS-15, Geriatric Depressive Symptoms Scale-15; GOS, galacto-oligosaccharide; HDL, high density lipoprotein; ICU, intensive care unit; IFABP, intestinal fatty acid binding protein; IFN, interferon; Ig, immunoglobulin; IL, interleukin; LPS, lipopolysaccharide; MMSE, Mini Mental State Examination; NK, natural killer; OS, oligosaccharide; RCT, randomized controlled trial; sCD14, soluble receptor CD14; SCFA, short-chain fatty acid; TNF, tumor necrosis factor; VAP, ventilator-associated pneumonia.

## The Role of GOS in Infectious Diseases, Inflammation, and Vaccine Efficacy

GOS has been proven effective in reducing the incidence and severity of infections, including respiratory infections in infants and children, rotavirus (RV)-associated diarrhea in early life, and acute diarrhea in adults, demonstrating its broad efficacy and safety across different age groups [[118], [119], [120]]. Given this broad efficacy, GOS presents a promising option for targeting the immunosenescent population, offering potential benefits in enhancing immune function in the elderly.

IBD is a chronic group of inflammatory conditions primarily affecting the gastrointestinal tract. The exact cause of IBD is not fully understood but is thought to involve genetic, environmental, and immune-related factors. Moreover, several factors, such as polypharmacy, comorbidities, treatment compliance, difficulties in differential diagnosis, and surgical complication risks often confound IBD in older adults [121] leading to their underrepresentation in clinical trials, which often exclude participants aged >65 y [122]. According to the European Crohn’s and Colitis Organization, 25% to 35% of patients with IBD are >60 y old [123]. Fiber consumption or Fermentable, Oligosaccharides, Disaccharides, Monosaccharides, and Polyols (FODMAP) diet is difficult to tolerate for patients with IBD or IBS, as it may exacerbate symptoms such as abdominal pain, discomfort, or diarrhea in specific individuals. However, 4 g of GOS supplementation twice daily did not induce intestinal bloating or abdominal discomfort in the elderly population [111]. In a study of adult mice fed 8.8, 4.4, and 2.2 g GOS/kg body weight showed an improvement in small bowel movements and relieved constipation [124]. Similarly, by increasing the quantity and activity of beneficial bacteria, GOS may benefit the gastrointestinal health of patients with IBD [96,125,126].

RV is a childhood infection that causes acute gastroenteritis. However, the rate of RV infection may be underestimated in the elderly population. In individuals aged ≥65 y, RV was detected in 9% of samples, indicating that RV infections could be more common in this age group than previously thought [127]. In one study, the combination of GOS and FOS induced an intestinal trophic effect and blocked RV infection in rats, thereby ameliorating RV-induced diarrhea [128]. An in vitro assay demonstrated the direct blocking interaction between GOS/FOS and RV, indicating that the virus was less detectable in the presence of the prebiotic mixture [129]. However, additional evidence and research are required to elucidate how GOS inhibits viral infections.

Vaccine efficacy can vary between individuals and is particularly low in older people [130]. Nagafuchi et al. [131] demonstrated that the seroprotective effect of an influenza vaccine was enhanced when fermented milk was administered in conjunction with GOS to H1N1-vaccinated, enterally-fed older individuals. A study showed that GOS improved Th1 responses and B cell activation specific to influenza vaccine in mice [132]. This suggests that the intake of prebiotics could be a practical approach to boost immune responses to the influenza vaccine. Given the immunomodulatory effect of GOS on gut homeostasis, researchers explored the role of GOS in preventing and treating age-related diseases. In a mouse study by Chen et al. [133], GOS reduced kidney inflammation by inhibiting the hypoxia-induced CD44/JNK cascade and cytokine production in renal tubular cells, thereby alleviating acute kidney injury, for which the risk is increased in individuals aged >75 y [134].

Animal and in vitro studies have provided robust evidence of the beneficial effect of GOS on the elderly immune system [124,128,129,132,133]. However, there is a lack of human clinical research in older adults, and most studies administered GOS in combination with other prebiotics (e.g., FOS, inulin) or probiotics, making it difficult to discern the individual effect of each component on the intervention. However, due to the combination of GOS with other prebiotics or probiotics in these trials, the immunomodulatory effects of GOS in the elderly lack robust evidence. Moreover, the effects of GOS supplementation on the immune system in older individuals are impacted by the GOS structures. For example, Maneerat et al. [135] reported no significant immunological or microbial changes in an aging cohort that received GOS that had predominantly Gal(β1–6), Gal(β1–4), and Gal(β1–3) linkages produced with the β-galactosidase from *Aspergillus oryzae* GC288. In contrast, another study showed that GOS consumption led to substantial increases in *Bacteroides* and *Bifidobacterium*, promoted the production of IL-10 and IL-8, and increased NK cell activity and C-reactive protein production in older subjects [17]. The GOS used in that study was produced by *Bifidobacterium bifidum* NCIMB 41171. These studies suggest that different sources of GOS may exert different immunomodulatory effects, and to better translate GOS into the clinical care of the elderly population and maximize their immunomodulatory effects, the dose, purity, and source of GOS need to be thoroughly investigated.

## Limitations of GOS Administration in the Elderly Population

The studies included in this review have reported the benefits of GOS supplementation in the short term (Table 1 [9,17,[111], [112], [113], [114], [115], [116], [117]]), but the long-term effects and sustainability of these benefits are less clear. The safety of GOS in infant formula and adult supplementation has been extensively researched, showing generally positive tolerability profiles with a No Observed Adverse Effect Level established at 5000 mg/kg body weight/d for 90 consecutive days and ≥2000 mg/kg/d [11]. In infants, GOSs are regularly added to formula at a concentration of 5 g/L, which has been shown to effectively enhance the beneficial gut microbiota without adverse effects [11,136]. The most commonly reported side effects among all age groups include bloating and digestive discomfort, which typically increase with higher doses of GOS [137]. For adults, studies have demonstrated that daily supplementation with GOS at doses from 5 to 20 g/d can be tolerated without significant side effects [138,139]. However, given the complicated health conditions in the elderly population (e.g. disease, lifestyle, immunosenescence), potential side effects and tolerability should be further investigated. Moreover, most of the clinical trials have used synbiotics or a mixture of other prebiotics with GOS as interventions in the elderly. The lack of studies administering GOS alone makes it difficult to apply in practice and introduces more variables that could affect the outcome. Also, as discussed earlier, the effectiveness of GOS can vary significantly from individual to individual, making it challenging to predict outcomes or standardize intervention. Although there is growing research on the benefits of GOS, there is still a lack of large-scale, long-term studies specifically focusing on the elderly population. Given these limitations, it is evident that comprehensive studies, including mechanistic and proof-of-concept research, are needed to provide a stronger rationale for utilizing GOS in elderly populations. Such studies would not only help in understanding the underlying mechanisms of how GOS benefits the elderly but also assist in developing standardized guidelines for their use, ensuring maximum efficacy and safety.

## Conclusions and Future Perspective

Recent advancements in our understanding of the direct and indirect modulatory effects of GOS have gained significant relevance due to the aging global population and the increasing prevalence of immune-related diseases in older adults. These developments underscore the importance of beneficial interventions in addressing age-related health challenges, with GOS as a potential intervention to prevent or counteract the deterioration of gut homeostasis associated with age. We summarized the health effects of GOS through the modulation of gut microbiota, enhancement of SCFAs and secondary bile acid production, and direct interactions with host receptors. This process contributes to the maintenance of a gut health state that is similar to younger individuals, which, in turn, can lead to an improved immune system and potentially increase longevity.

The positive clinical outcomes associated with GOS suggest that it could be a feasible approach to support immune health and address the health challenges associated with aging. Nevertheless, it is important to recognize that the physiological and immunological alterations unique to the aging process require further targeted research. Such research should focus specifically on the older adult population to ensure that interventions like GOS supplementation are safe, effective, and tailored to meet the unique needs of this demographic.

## Author contributions

The authors’ responsibilities were as follows—YH, MAA-P: wrote the manuscript, writing - original draft; MRA: writing - review and editing and all authors: read and approved the final manuscript.

## Conflict of interest

The authors report no conflicts of interest.
